# Impact of older age in patients receiving atezolizumab and bevacizumab for hepatocellular carcinoma

**DOI:** 10.1111/liv.15405

**Published:** 2022-09-02

**Authors:** Mathew Vithayathil, Antonio D'Alessio, Claudia A. M. Fulgenzi, Naoshi Nishida, Martin Schönlein, Johann von Felden, Kornelius Schulze, Henning Wege, Anwaar Saeed, Brooke Wietharn, Hannah Hildebrand, Linda Wu, Celina Ang, Thomas U. Marron, Arndt Weinmann, Peter R. Galle, Dominik Bettinger, Bertram Bengsch, Arndt Vogel, Lorenz Balcar, Bernhard Scheiner, Pei‐Chang Lee, Yi‐Hsiang Huang, Suneetha Amara, Mahvish Muzaffar, Abdul Rafeh Naqash, Antonella Cammarota, Nicola Personeni, Tiziana Pressiani, Matthias Pinter, Alessio Cortellini, Masatoshi Kudo, Lorenza Rimassa, David J. Pinato, Rohini Sharma

**Affiliations:** ^1^ Department of Surgery & Cancer, Imperial College London Hammersmith Hospital London UK; ^2^ Department of Biomedical Sciences Humanitas University Pieve Emanuele Italy; ^3^ Division of Medical Oncology Policlinico Universitario Campus Bio‐Medico Rome Italy; ^4^ Department of Gastroenterology and Hepatology Kindai University Faculty of Medicine Osaka Japan; ^5^ Department of Oncology, Hematology and Bone Marrow Transplantation with Section of Pneumology University Medical Center Hamburg‐Eppendorf Hamburg Germany; ^6^ Department of Medicine University Medical Center Hamburg‐Eppendorf Hamburg Germany; ^7^ Division of Medical Oncology, Department of Medicine Kansas University Cancer Center Kansas City Kansas USA; ^8^ Division of Hematology/Oncology, Department of Medicine Tisch Cancer Institute, Mount Sinai Hospital New York New York USA; ^9^ I. Medical Department University Medical Center Mainz Mainz Germany; ^10^ Department of Medicine II (Gastroenterology, Hepatology, Endocrinology and Infectious Diseases), Faculty of Medicine, Freiburg University Medical Center University of Freiburg Freiburg Germany; ^11^ University of Freiburg Signalling Research Centers BIOSS and CIBSS Freiburg Germany; ^12^ German Cancer Consortium (DKTK), Partner Site Freiburg Germany; ^13^ Hannover Medical School Hannover Germany; ^14^ Division of Gastroenterology & Hepatology, Department of Internal Medicine III Medical University of Vienna Vienna Austria; ^15^ Division of Gastroenterology and Hepatology, Department of Medicine Taipei Veterans General Hospital Taipei Taiwan; ^16^ Institute of Clinical Medicine, School of Medicine National Yang Ming Chiao Tung University Taipei Taiwan; ^17^ Division of Hematology/Oncology East Carolina University Greenville North Carolina USA; ^18^ Medical Oncology/TSET Phase 1 Program, Stephenson Cancer Center University of Oklahoma Norman Oklahoma USA; ^19^ Medical Oncology and Hematology Unit, Humanitas Cancer Center IRCCS Humanitas Research Hospital Rozzano Italy; ^20^ Division of Oncology, Department of Translational Medicine University of Piemonte Orientale Novara Italy

**Keywords:** anti‐programmed death‐ligand, anti‐vascular endothelial growth factor, checkpoint inhibitor, cirrhosis, immunotherapy

## Abstract

**Background and Aims:**

Combination atezolizumab/bevacizumab is the gold standard for first‐line treatment of unresectable hepatocellular carcinoma (HCC). Our study investigated the efficacy and safety of combination therapy in older patients with HCC.

**Methods:**

191 consecutive patients from eight centres receiving atezolizumab and bevacizumab were included. Overall survival (OS), progression‐free survival (PFS), overall response rate (ORR) and disease control rate (DCR) defined by RECIST v1.1 were measured in older (age ≥ 65 years) and younger (age < 65 years) age patients. Treatment‐related adverse events (trAEs) were evaluated.

**Results:**

The elderly (*n* = 116) had higher rates of non‐alcoholic fatty liver disease (19.8% vs. 2.7%; *p* < .001), presenting with smaller tumours (6.2 cm vs 7.9 cm, *p* = .02) with less portal vein thrombosis (31.9 vs. 54.7%, *p* = .002), with fewer patients presenting with BCLC‐C stage disease (50.9 vs. 74.3%, *p* = .002). There was no significant difference in OS (median 14.9 vs. 15.1 months; HR 1.15, 95% CI 0.65–2.02 *p* = .63) and PFS (median 7.1 vs. 5.5 months; HR 1.11, 95% CI 0.54–1.92; *p* = .72) between older age and younger age. Older patients had similar ORR (27.6% vs. 20.0%; *p* = .27) and DCR (77.5% vs. 66.1%; *p* = .11) compared to younger patients. Atezolizumab‐related (40.5% vs. 48.0%; *p* = .31) and bevacizumab‐related (44.8% vs. 41.3%; *p* = .63) trAEs were comparable between groups. Rates of grade ≥3 trAEs and toxicity‐related treatment discontinuation were similar between older and younger age patients. Patients 75 years and older had similar survival and safety outcomes compared to younger patients.

**Conclusions:**

Atezolizumab and bevacizumab therapy is associated with comparable efficacy and tolerability in older age patients with unresectable HCC.

**Abbreviations**
AASLD: American Association for the Study of Liver Diseases
AFP: alpha‐fetoprotein
BCLC: Barcelona Clinic Liver Cancer
CR: complete response
CI: confidence interval
CTCAE: National Cancer Institute Common Terminology Criteria for Adverse Events
DCR: disease control rate
ECOG‐PS: Eastern Cooperative Oncology Group Performance Status
HCC: hepatocellular carcinoma
HR: hazard ratio
ORR: overall response rate
OS: overall survival
PD‐L1: programmed death ligand
PFS: progression‐free survival
PR: partial response
PD: progressive disease
MTD: maximal tumour diameter
MVI: macrovascular invasion
NAFLD: non‐alcoholic fatty liver disease
NSCLC: non‐small cell lung cancer
SD: stable disease
SmPC: summary of product characteristics
TACE: trans‐arterial chemoembolization
trAE: treated‐related adverse event
VEGF: vascular endothelial growth factor

**Lay Summary**

Atezolizumab and bevacizumab are effective cancer treatments for patients with liver cancer. However, its effectiveness and safety in older patients with liver cancer are not as clear. Our study demonstrates this treatment is effective in older patients, without an increase in side effects.

## 1 BACKGROUND

Hepatocellular carcinoma (HCC) is the fourth leading cause of cancer mortality and the sixth most prevalent worldwide.^1^ Until recently, systemic multikinase inhibitors including sorafenib^2^ and lenvatinib^3^ were the mainstay of unresectable HCC. However, the IMbrave150 study investigating combination therapy with atezolizumab, anti‐programmed death‐ligand (PD‐L1) and bevacizumab, anti‐vascular endothelial growth factor (VEGF) showed superiority over sorafenib for both overall survival (OS) and progression‐free survival (PFS).^4^, ^5^ In unresectable HCC patients, combination therapy improved median OS of 19.2 vs. 13.4 months (hazard ratio (HR) 0.66, 95% confidence interval (CI) 0.52 to 0.85) and median PFS to 6.9 vs. 4.3 months (HR 0.65, 95% CI 0.53 to 0.81). Atezolizumab and bevacizumab now represent a first‐line treatment option in unresectable HCC, alongside a combination of tremelimumab and durvalumab.^6^

Increasing age is an established risk factor for HCC.^7^ Curative therapies such as surgical resection^8^, ^9^ and local ablation^10^, ^11^ have shown favourable outcomes in older patients. However, due to increasing co‐morbidities with increasing age, lower rates of curative therapies in older patients are observed.^9^ Therefore, understanding the efficacy and safety of non‐curative therapy in these patients is paramount. Studies have shown that trans‐arterial chemoembolization (TACE)^12^ and systemic therapies such as sorafenib^13^ and cabozantinib^14^ are beneficial in older patients without an increase in adverse events and drug toxicity.^15^, ^16^

The impact of immunotherapy in older age patients is less well studied than in younger cohorts, due to an underrepresentation in cancer trials.^17^, ^18^, ^19^ Previous studies assessing the efficacy and safety of immunotherapy in older patients demonstrate favourable results. A meta‐analysis of 17 randomized control trials in patients receiving nivolumab, pembrolizumab or atezolizumab for metastatic solid organ tumours showed patients aged 65 and over had similar OS and PFS compared to patients under 65.^20^ Similarly, another meta‐analysis of 5265 patients showed immunotherapy was efficacious in both older and younger patients across multiple cancer sites,^21^ results supported by a number of other studies across different cancer sites.^22^, ^23^, ^24^ Subgroup analysis from the IMbrave150 trial demonstrated an increased overall and progression‐free survival for combination of atezolizumab/bevacizumab compared to sorafenib in patients aged 65 or older, in line with the results of the entire cohort, with similar safety profiles seen in the elderly.^25^

Our retrospective study aimed to evaluate the real‐world efficacy and safety of combination atezolizumab and bevacizumab in older age patients for unresectable HCC.

## 2 METHODS AND MATERIALS

### 2.1 Study participants and design

This was a multi‐centre retrospective cohort study. Study participants were consecutive patients with unresectable HCC receiving atezolizumab plus bevacizumab across eight tertiary centres in Germany, Japan, Austria, United Kingdom, Italy, Taiwan and United States of America. All patients had a histological or radiological diagnosis of HCC in accordance with American Association for the Study of Liver Diseases (AASLD) criteria^26^ and advanced disease or intermediate disease unsuitable or refractory to locoregional therapies, as per the Barcelona Clinic Liver Cancer (BCLC) criteria.^27^ In patients with chronic liver disease, aetiology of liver disease was defined using the European Association for the Study of the Liver (EASL) guidelines. Patients receiving previous systemic cancer therapy were excluded. Patients with Child–Pugh class B liver dysfunction were included in the study, based on previous safety and efficacy of immunotherapy in this cohort.^28^, ^29^ All patients received combination atezolizumab plus bevacizumab in accordance with the IMbrave150 protocol: atezolizumab 1200 mg and bevacizumab 15 mg/kg intravenously every 3 weeks. Dosing modification and toxicity management were conducted by local treating teams. Decisions for treatment discontinuation due to disease progression and/or unacceptable toxicity was made by multi‐disciplinary assessment at each local centre.

### 2.2 Patient outcomes

Patients' baseline demographics and clinical parameters including underlying liver disease aetiology, Child–Pugh class, BCLC stage, Eastern Cooperative Oncology Group Performance Status (ECOG‐PS) were collected. Overall survival (OS) was defined as the time in months from first drug administration to date of death or date of last follow‐up. Profession‐free survival (PFS) was the time from first drug administration to date of progression on radiological imaging or death whichever came first. Treatment response was evaluated using RECIST criteria v1.1 on CT or MRI at 9–12 week intervals.^30^ Overall response rate (ORR) included all patients with complete response (CR) and partial response (PR). Disease control rate (DCR) included all patients with CR, PR or stable disease (SD). Progressive disease (PD) included all patients with radiological evidence of intra‐ or extra‐hepatic spread. Treatment‐related adverse events (trAEs) for atezolizumab and bevacizumab were defined as per the summary of product characteristics (SmPC). TrAEs were graded as per the National Cancer Institute Common Terminology Criteria for Adverse Events (CTCAE) v. 5.0.^31^

### 2.3 Statistical analysis

For age analysis, patients 65 years or older were classified as older age as per classification from United Nations^32^ and previous age‐related immunotherapy meta‐analysis,^20^, ^21^, ^24^ including subgroup analysis of the IMbrave150 trial.^25^ Those aged less than 65 years are classified as younger age. Baseline characteristics in each cohort were compared using the χ^2^ test for categorical data, and the unpaired Student's *t*‐test for continuous data. The proportion of emerging TrAEs and ORR/DCR were compared between age cohorts using the χ^2^ test.

Time‐to‐event analysis was performed for OS and PFS using the Kaplan–Meier method. OS and PFS were compared between age cohorts using log rank. A *p* value of less than .05 was defined as statistically significant. Univariate and multivariate Cox regression models for older age and established prognostic factors were conducted for OS and PFS.

Due to the increasing age and life expectancy of patients, survival and safety analysis was performed using patients 75 years and older and patients under 75 years old.

### 2.4 Ethics Statement

This study received ethical approval by Imperial College Tissue Bank (Reference Number R16008) and by the local ethics committee of each treating centre.

## 3 RESULTS

### 3.1 Baseline characteristics

There were 210 patients receiving atezolizumab and bevacizumab from the 12 centres between January 2020 and December 2021. Nineteen patients had received prior systemic therapy and were excluded from the study. The baseline characteristics of the 191 consecutive patients receiving atezolizumab plus bevacizumab are shown in Table 1. Forty‐four patients had Child–Pugh B cirrhosis, with viral hepatitis the most prevalent cause of the underlying chronic liver disease (57.1%). Extrahepatic disease was present in 37.7% of patients.

**TABLE 1:** Baseline characteristics of the study population stratified by age

	All patients (*n* = 191)	Younger age (*n* = 75)	Older age (*n* = 116)	*p‐*value
Centre				
Germany	30 (15.7)	10 (13.3)	20 (17.2)	<.001
Austria	12 (6.3)	2 (2.7)	10 (8.6)	
United Kingdom	15 (7.9)	9 (12.0)	6 (5.2)	
Italy	12 (6.3)	7 (9.3)	5 (4.3)	
United States of America	60 (31.4)	32 (42.7)	28 (24.1)	
Japan	51 (26.7)	8 (10.7)	43 (37.1)	
Taiwan	11 (5.8)	7 (9.3)	4 (3.5)	
Median age (IQR, years)	68.4 (61.8–75.2)	59.5 (52.6–62.9)	73.2 (69.7–79.0)	<.001
Male sex	161 (84.3)	66 (88.0)	95 (81.9)	.21
Risk factors for chronic liver disease				
Non‐alcoholic fatty liver disease	25 (13.1)	2 (2.7)	23 (19.8)	<.001
Alcohol related	73 (38.2)	28 (37.3)	45 (38.8)	.84
Hepatitis B infection	37 (19.4)	20 (26.7)	17 (14.7)	.04
Hepatitis C infection	72 (37.7)	34 (45.3)	38 (32.8)	.08
Other	12 (8.6)	4 (6.0)	8 (11.0)	.29
Child–Turcotte–Pugh class				
A	147 (77.0)	56 (74.7)	91 (78.5)	.54
B	44 (23.0)	19 (25.3)	25 (21.6)	
Baseline liver disease				
Ascites	57 (29.8)	22 (29.3)	35 (30.2)	.90
Hepatic encephalopathy	11 (5.8)	9 (12.0)	2 (1.7)	.003
Varices present	39 (20.4)	20 (26.7)	19 (16.4)	.09
Maximum tumour diameter (cm)	6.8 (4.9)	7.9 (5.0)	6.2 (4.7)	.02
Macrovascular invasion (MVI)	78 (40.8)	41 (54.7)	37 (31.9)	.002
Extrahepatic spread (EHS)	72 (37.7)	31 (41.3)	41 (35.3)	.40
AFP (ng/dl)				
≤400	126 (66.0)	49 (65.3)	77 (66.4)	.88
>400	65 (34.0)	26 (34.7)	39 (33.6)	
ECOG‐PS				
0	119 (63.0)	45 (60.8)	74 (64.4)	.67
1	64 (33.9)	25 (33.8)	39 (33.9)	
2	6 (3.2)	4 (5.4)	2 (1.7)	
Barcelona clinic liver cancer stage				
A	7 (3.7)	0	7 (6.1)	.002
B	68 (36.2)	19 (25.7)	49 (43.0)	
C	113 (60.1)	55 (74.3)	58 (50.9)	
ALBI score	−2.2 (0.6)	−2.2 (0.6)	−2.3 (0.6)	.77
Grade 1	67 (35.1)	24 (32.0)	43 (37.1)	.47
Grade 2	106 (55.5)	45 (60.0)	61 (52.6)	.31
Grade 3	18 (9.4)	6 (8.0)	12 (10.3)	.59
Laboratory				
Serum albumin (g/L)	35.8 (5.9)	36.0 (5.4)	35.7 (6.2)	.69
Bilirubin (μmol/L)	23.1 (40.9)	23.2 (19.8)	23.1 (50.1)	.99
Platelet count (x10^9^/L)	181.7 (97.9)	185 (106.9)	179.5 (91.9)	.71
Previous locoregional treatment				
Resection	44 (23.0)	12 (16.0)	32 (27.6)	.17
Radiofrequency ablation	38 (19.9)	9 (12.0)	29 (25.0)	.02
Transarterial chemoembolization	57 (29.8)	19 (25.3)	38 (32.8)	.27
Y90	21 (11.0)	7 (9.3)	14 (12.1)	.56
External beam radiotherapy	6 (3.1)	0	6 (5.2)	.045
Median immunotherapy duration (IQR, months)	3.5 (1.5–7.7)	3.5 (1.6–6.6)	3.4 (1.4–8.1)	.42

*Note*: *n* (%) for discrete variables; mean ± standard deviation for continuous variables.

Abbreviations: AFP, alpha‐fetoprotein; ECOG‐PS, Eastern Cooperative Oncology Group Performance Status.

One hundred and sixteen patients (60.7%) were 65 years or older when receiving the first dose of atezolizumab plus bevacizumab, and 75 (39.3%) were less than 65 years old. The older cohort had a higher proportion of non‐alcoholic fatty liver disease (NAFLD) (19.8% vs. 2.7%; *p* < .001), with a lower rate of chronic Hepatitis B infection (14.7% vs 26.7%; *p* = .04). Older patients had smaller tumours (maximal tumour diameter [MTD] 6.2 cm vs 7.9 cm, *p* = .02), an inferior proportion of macrovascular invasion (MVI) (31.9 vs 54.7%, *p* = .002) and were more likely to commence treatment with BCLC‐B stage disease (43.0 vs 25.7%, *p* = .002). Older patients had similar rates of ascites (30.2% vs. 29.3%; *p* = .90) and varices (16.4% vs. 26.7%; *p* = .09), but lower rates of hepatic encephalopathy (1.7% vs. 12.0%; *p* = .003). Rates of Child–Pugh B cirrhosis were similar between the age groups (21.6% vs. 25.3%; *p* = .54).

### 3.2 Efficacy

**FIGURE 1:**
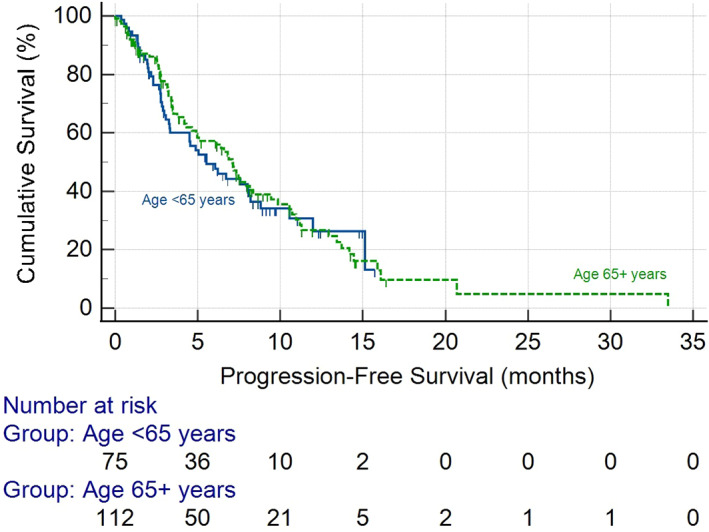
Kaplan–Meier curve showing overall survival for older age and younger age patients.

**FIGURE 2:**
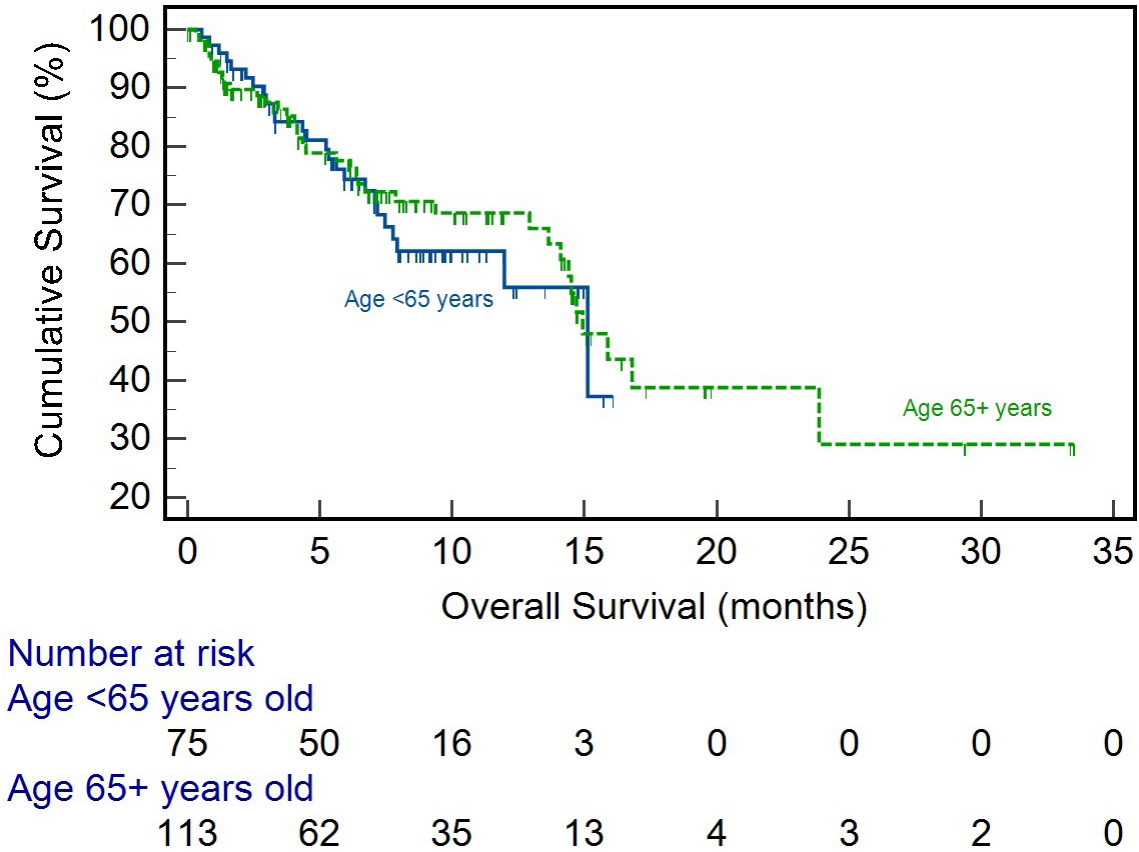
Kaplan–Meier curve showing progression‐free survival for older age and younger age patients.

The median duration of treatment with atezolizumab and bevacizumab was 3.5 months (interquartile range [IQR] 1.5–7.7 months). Treatment duration was comparable between the older and younger groups (3.4 (IQR 1.4–8.1) months vs. 3.5 (IQR 1.6–6.6) months; *p* = .42). At the time of analysis 62 (32.5%) patients had died. Survival was comparable between the age cohorts with older age having a median survival of 14.9 months compared to 15.1 months (*p* = .67) for those <65 years (Figure 1). Older age did not have a significant effect on OS in univariate (hazard ratio (HR) 0.65, 95% confidence interval (CI) (0.53–1.49), *p* = .65) and multivariate (HR 1.15, 95% CI 0.65–2.02, *p* = .63) analysis (Table 2). Median PFS was also comparable between the age cohorts (7.1 vs 5.5 months, *p* = .69) (Figure 2), with no effect of age on PFS seen in multivariate analysis (HR 1.11, 95% CI 0.54–1.92, *p* = .72) (Table S1). Disease response was assessed in 163 patients as per RECIST v1.1 criteria. Older age patients had similar ORR (25.6% vs. 20.0%; *p* = .27) and DCR (77.5% vs.66.1%; *p* = .11) compared to younger patients (Table 3).

**TABLE 2:** Effects of older age and prognostic factors on overall survival after atezolizumab and bevacizumab in univariate and multivariate Cox regression models

	Univariate models		Multivariable models	
	Hazard ratio (95% CI)	*p‐*value	Hazard ratio (95% CI)	*p‐*value
Age ≥ 65 years	0.65 (0.53–1.49)	.65	1.15 (0.65–2.02)	.63
BCLC Stage (C vs A or B)	1.50 (0.89–2.52)	.13	0.98 (0.53–1.82)	.96
CTP Class (B vs A)	3.01 (1.77–5.13)	<.001	2.51 (1.39–4.54)	.002
Tumour size >7 cm	1.30 (0.77–2.20)	.32	1.15 (0.66–1.99)	.62
MVI	2.51 (1.15–4.18)	<.001	1.88 (1.00–3.54)	.05
Metastatic disease	0.80 (0.47–1.36)	.41	0.94 (0.54–1.66)	.84
AFP > 400 ng/dl	1.32 (0.79–2.19)	.29	1.17 (0.69–1.99)	.56
HCV vs other aetiologies	1.51 (0.91–2.50)	.11	1.74 (1.03–2.94)	.04

Abbreviations: 95% CI, 95% Confidence Interval; AFP, alpha‐fetoprotein; BCLC, Barcelona Clinic Liver Cancer; CTP, Child–Turcotte–Pugh; HCV, Hepatitis C virus; MVI, Macrovascular invasion.

**TABLE 3:** Best radiological response evaluated per RECIST criteria version 1.1 stratified by age

	All patients (*n* = 163^a^)	Younger age (*n* = 65)	Older age (*n* = 98)	*p*‐value
Complete response	0	0	0	
Partial response	40 (24.5)	13 (20.0)	27 (27.6)	.27
Stable disease	79 (48.5)	30 (46.2)	49 (50.0)	.63
Progressive disease rate	44 (27.0)	22 (33.9)	22 (22.5)	.11

Abbreviation: RECIST, response evaluation criteria in solid tumours.

^a^ Radiological response was assessed in 163 patients (85.3%).

As Child–Pugh class had a significant effect on OS and PFS in multivariate survival analysis, we performed subgroup analysis in patients by Child–Pugh class. Older patients had comparable OS (median OS 6.2 months, 95% CI 4.1–15.9 vs. 5.9 months, 95% CI 3.0–15.1; *p* = .80) and PFS (median PFS 3.7 months, 95% CI 3.2–7.5 vs. 3.3 months, 95% CI 2.3–15.1; *p* = .97) in patients with Child–Pugh B liver disease. Similar comparable survival outcomes were seen in patients with Child–Pugh A disease for older patients.

### 3.3 Safety

There was no difference in trAEs of any grade between older and younger age patients (62.1% vs. 73.3%; *p* = .11) (Table 4). There was no difference in grade 3 or higher AEs between the age cohorts (20.7% vs. 20.0%; *p* = .91). Rates of atezolizumab‐related AEs were comparable between the two groups, the most common being fatigue in both age cohorts (13.8% vs. 20.0%; *p* = .26). Bevacizumab‐related trAEs were similar between the two groups (44.8% vs. 41.3, *p* = .63). No difference was noted in rates of treatment discontinuation with seven patients 65 years and older and five patients under 65 discontinuing treatment due to trAEs (6.0% vs. 6.7%; *p* = .86). There was no difference in grade 3 or higher atezolizumab‐ and bevacizumab‐related trAEs between age.

**TABLE 4:** Atezolizumab and bevacizumab treatment‐related adverse events stratified by age

	All patients (*n* = 191)	Younger age (*n* = 75)	Older age (*n* = 116)	*p‐*value
Any grade trAEs	127 (66.5)	55 (73.3)	72 (62.1)	.11
Grade ≥ 3^a^ trAEs	39 (20.4)	15 (20.0)	24 (20.7)	.91
Atezolizumab‐related	15 (7.9)	7 (9.3)	8 (6.9)	.54
Bevacizumab‐related	26 (13.6)	9 (12.0)	17 (14.7)	.60
trAEs requiring drug discontinuation	12 (6.3)	5 (6.7)	7 (6.0)	.86
Atezolizumab trAEs				
Overall	83 (43.5)	36 (48.0)	47 (40.5)	.31
Fatigue	31 (16.2)	15 (20.0)	16 (13.8)	.26
Hepatotoxicity	28 (14.7)	13 (17.3)	15 (12.9)	.41
Skin toxicity	9 (4.7)	3 (4.0)	6 (5.2)	.71
Colitis	24 (12.6)	7 (9.3)	17 (14.7)	.28
Thyroid dysfunction	9 (4.7)	3 (4.0)	6 (5.2)	.71
Pneumonitis	4 (2.1)	1 (1.3)	3 (2.6)	.55
Bevacizumab trAEs				
Overall	83 (43.5)	31 (41.3)	52 (44.8)	.63
Bleeding	20 (10.5)	11 (14.7)	9 (7.8)	.13
Hypertension	44 (23.0)	13 (17.3)	31 (26.7)	.13
Proteinuria	38 (19.9)	13 (17.3)	25 (21.6)	.48
Thrombosis	10 (5.2)	4 (5.3)	6 (5.2)	.96

Abbreviation: trAE, treatment‐related adverse event.

^a^ Graded as per the National Cancer Institute Common Terminology Criteria for Adverse Events (CTCAE).

### 3.4 Cohorts

#### 3.4.1 Patients aged 75 years and older

Given the rising age and life expectancy survival and safety analysis was performed using an age‐cut off of 75 years old (Table S2). Patients aged 75 years and older had a similar median OS (14.7 months, 95% CI 13.6–15.9 vs. 15.1 months, 95% CI 12.9–23.9; *p* = .90) (Figure S1) and median PFS (4.9 months, 95% CI 3.2–11.2 vs. 6.8 months, 95% CI 4.9–8.2; *p* = .76) (Figure S2), with no difference in radiological response (Table S5). Age 75 years and older did not affect OS and PFS in multivariate analysis (Tables S3 and S4). These patients did not have higher rates of trAEs or trAE‐related drug discontinuation compared to younger patients (Table S6).

## 4 DISCUSSION

The landmark IMbrave150 study resulted in a step change in the management of advanced HCC. Combination atezolizumab/bevacizumab is now a recommended first‐line therapy in patients who have no contraindication to either drug. Recent results from the HIMALAYA trial demonstrate tremelimumab and durvalumab superiority to monotherapy in unresectable HCC, further demonstrating the efficacy of combination immunotherapy.^6^ The median age of the population studied in IMbrave150 was 64^4^ whilst the peak age of HCC incidence in the western world is between 80 and 85,^33^ highlighting the underrepresentation of elderly patients in clinical trials. We conducted an international study to investigate the efficacy and safety of atezolizumab plus bevacizumab in patients ≥65 years compared to a younger age group. We demonstrated that older age patients have similar survival and disease control rates compared to younger patients. Moreover, there was no difference in the incidence of adverse events between the two age groups. We observed similar efficacy and safety profiles in patients 75 years and older. To the best of our knowledge, this is the first study to date to investigate the real‐world safety and efficacy of atezolizumab plus bevacizumab for older patients with HCC.^15^, ^16^

A recent subgroup analysis of the IMbrave150 investigated the efficacy and safety of atezolizumab/bevacizumab in unresectable HCC patients aged 65 years and older.^25^ Combination therapy maintained superior OS (HR 0.58, 95% CI 0.36–0.92) and PFS (0.63, 95% CI 0.45–0.89) to sorafenib in elderly patients. We report a comparable median PFS (7.1 months vs. 7.7 months) and ORR (27.6% vs. 26%) to the IMbrave150 study in the older cohort. The authors observed a higher proportion of females and lower baseline AFP in the older group compared to the younger cohort, which we did not observe. These differences may relate to differences in sample size. Our older cohort had a smaller MTD, higher rate of MVI and was more likely to present with earlier stage disease compared to the younger patients. In a large, multicentre cohort study of 1068 patients, patients older patients with HCC were more likely to be female and were less likely to have multinodular cancers and less MVI.^34^ Similar to our study, the authors report that age per se did not impact on survival outcomes.

Two large meta‐analyses have reviewed the efficacy of immunotherapy in older patients with non‐HCC cancer types. Elias et al. reviewed 17 randomized controlled trials using nivolumab, pembrolizumab and atezolizumab.^20^ 2324 patients over 65 years old had a similar benefit in OS and PFS to young patients receiving immunotherapy for head and neck cancer, melanoma, non‐small cell lung cancer (NSCLC) and renal cell carcinoma. These findings were consistent with another meta‐analysis showing older patients had improved survival after immunotherapy (HR 0.73, 95% CI 0.62–0.87; *p* < .001) compared to systemic chemotherapy.^21^ Furthermore, a large retrospective analysis of 18 international centres showed immunotherapy was effective in 928 patients over the age of 80 years across NSCLC, melanoma and genitourinary tumours.^22^ Our results are consistent with these previous findings and demonstrate immunotherapy confers benefit in older patients with HCC.

In our study, there was a higher proportion of underlying NAFLD in the elderly cohort, and a lower proportion of Hepatitis C compared to the younger age group. A recent meta‐analysis showed that non‐viral HCC may not fully benefit from improved survival after immunotherapy,^35^ suggesting underlying liver aetiology may influence treatment response, an area of active research. However, despite a higher proportion of non‐viral HCC in the older age cohort, we observed OS and PFS did not diminish compared to the younger age group. We observed that CP‐B cirrhosis was significantly associated with reduced OS and PFS in multivariate analysis. D'Alessio et al.^28^ previously reported real‐world use of atezolizumab/bevacizumab in patients with CP‐B liver dysfunction, and though studies report safe use of immunotherapy in decompensated liver disease,^29^ its impact on efficacy is not yet clear.

A concern for the use of cancer therapy in older age patient is significant toxicity. Systemic chemotherapy has been associated with higher rates of trAEs,^36^ treatment‐related mortality^37^ and treatment discontinuation^38^ in older patients. Studies evaluating the safety of immunotherapy in this population are more limited. In metastatic melanoma, older patients did not show increased rates of overall trAEs; though higher rates of arthritis^39^ and endocrine‐related^40^ toxicity were observed. Increased rate of hypertension with bevacizumab in the elderly was previously observed by Hurwitz and colleagues when investigating the addition of bevacizumab to chemotherapy for the management of colorectal cancer^41^ but no differences in rates of proteinuria have been previously reported in other tumour types.^42^, ^43^ In our study, we did not observe a difference in atezolizumab or bevacizumab‐related trAEs between age groups. This is in line with the IMbrave150 analysis, which did not show a higher rate of trAEs in the older cohort, despite higher rates of baseline comorbidities such as hypertension, diabetes mellitus and hyperlipidaemia suggesting immunotherapy is safe in the older patient group.^25^

The relationship between ageing, cancer and immunity is complex. Immune function has been shown to decline with increasing age^44^, ^45^ and has been proposed as a driver of increasing cancer risk.^46^ This immunosenescence has led to concerns immunotherapy may not be effective in older patients. However, Kugel et al. demonstrated downregulation of regulatory T cells in older mice with melanoma.^47^ These older mice showed a greater response to anti‐PD1 therapy compared to younger mice, with blockage of regulatory T cells also increase response. A decrease in regulatory T cells with older age may potentiate the response to immunotherapy. Further understanding of the interplay between ageing, immunity and cancer may guide the choice of immunotherapy in older patients.

Our study has some limitations. This is a retrospective study, and therefore subject to collection and selection bias. Across eight tertiary centres, there may be inter‐site variation in treatment protocols, follow‐up, efficacy and safety assessments. We chose an age of 65 years as a cut‐off between the two groups. This was based on boundaries used in previous large meta‐analyses,^20^, ^21^, ^24^ previous IMbrave age subgroup analysis^25^ and definitions from the United Nations.^32^ However, there are varying definitions of older age, influenced by additional factors such as medical co‐morbidities and frailty.^48^ We report all‐cause mortality, rather than liver‐specific mortality, which may be impacted by unreported medical comorbidities. Despite these limitations, to the best of our knowledge, this is the largest study assessing efficacy and safety for real‐time use of atezolizumab plus bevacizumab for HCC in an older age cohort.

## 5 CONCLUSION

Our study shows atezolizumab and bevacizumab therapy to be efficacious and safe to use in older patients with unresectable HCC. Clinicians should not be deterred in administering combination therapy in older patients, provided eligibility for combination immunotherapy is otherwise met.

## CONSENT FOR PUBLICATION

All authors consented to the publication of the manuscript.

## FUNDING INFORMATION

MV is supported by the National Institute of Health Research. AD is supported by the NIHR Imperial BRC and by grant funding from the European Association for the Study of the Liver (Andrew Burroughs Fellowship) and from Cancer Research UK (RCCPDB‐Nov21/100008). DJP is supported by grant funding from the Wellcome Trust Strategic Fund (PS3416) and from the Associazione Italiana per la Ricerca sul Cancro (AIRC MFAG Grant ID 25697). We acknowledge support from the NIHR Imperial Biomedical Research Centre (BRC), the Imperial Experimental Cancer Medicine Centre (ECMC) and the Imperial College Tissue Bank.

## CONFLICT OF INTEREST

AD received educational support for congress attendance from Roche. JvF received advisory board fees from Roche. HW received lecture fees and advisory board honoraria from Roche, Bayer, Ipsen, Eisai, BMS. AS received research grants (to institution) from AstraZeneca, Merck, Bristol Myers Squibb, Exelixis, Clovis, KAHR medical, Actuate therapeutics, Incyte Corp. and Advisory board fees from AstraZeneca, Bristol Myers Squibb, Merck, Exelixis and Pfizer. PRG reports a consulting or advisory role and received honoraria from AdaptImmune, AstraZeneca, Bayer, Bristol Myers Squibb, Eisai, Ipsen, Lilly, Merck Sharp & Dohme, Roche and Sirtex; has been on a speakers bureau for straZeneca, Bayer, Bristol Myers Squibb, Eisai, Ipsen, Lilly, Merck Sharp & Dohme, Roche and Sirtex; has received research funding from Bayer and Roche; has provided expert testimony for Lilly; and has received travel or accommodation expenses from AstraZeneca, Bayer, Bristol Myers Squibb, Eisai, Ipsen, Lilly and Roche. DB has received lecture and speaker fees from Bayer Healthcare, the Falk Foundation Germany and consulting fees from Boston Scientific. AV reports honoraria for speaker, consultancy and advisory role from Roche, AstraZeneca, EISAI, Bayer, Merck, Bristol Myers Squibb, Merck Sharp & Dohme, Incyte, PierreFabre, Ipsen and Sanofi. BS received travel support from Gilead, Ipsen and AbbVie. NP received consulting fees from Amgen, Merck Serono, Servier; lectures fees from AbbVie, Gilead, Lilly, Sanofi; travel expenses from Amgen, ArQule; and institutional research funding from Basilea, Merck Serono, Servier. TP received consulting fees from Bayer; and institutional research funding from Bayer, Lilly, Roche. RS received consulting fees for EISAI, Roche, Bayer, SIRTEX, Novartis; research funding (to institution) from Incyte, Novartis, Astex Pharmaceuticals, Bayer and Boston Scientific. MP is an investigator for Bayer, BMS, Ipsen, Lilly and Roche; he received speaker honoraria from Bayer, BMS, Eisai, Lilly, MSD and Roche; he is a consultant for Bayer, BMS, Eisai, Ipsen, Lilly, MSD and Roche; he received travel support from Bayer and BMS. AC received consulting fees from MSD, BMS, AstraZeneca, Roche; speakers' fee from AstraZeneca, MSD, Novartis and Astellas. LR received consulting fees from Amgen, ArQule, AstraZeneca, Basilea, Bayer, BMS, Celgene, Eisai, Exelixis, Genenta, Hengrui, Incyte, Ipsen, IQVIA, Lilly, MSD, Nerviano Medical Sciences, Roche, Sanofi, Servier, Taiho Oncology, Zymeworks; lecture fees from AbbVie, Amgen, Bayer, Eisai, Gilead, Incyte, Ipsen, Lilly, Merck Serono, Roche, Sanofi; travel expenses from Ipsen and AstraZeneca; and institutional research funding from Agios, ARMO BioSciences, AstraZeneca, BeiGene, Eisai, Exelixis, Fibrogen, Incyte, Ipsen, Lilly, MSD, Nerviano Medical Sciences, Roche, Zymeworks. DJP received lecture fees from ViiV Healthcare, Bayer Healthcare, BMS, Roche, Eisai, Falk Foundation, travel expenses from BMS and Bayer Healthcare; consulting fees for Mina Therapeutics, EISAI, Roche, DaVolterra, Mursla, Exact Sciences and Astra Zeneca; research funding (to institution) from MSD and BMS. All remaining authors have declared no conflicts of interest. The authors have no other relevant affiliations or financial involvement with any organization or entity with a financial interest in or financial conflict with the subject matter or materials discussed in the manuscript apart from those disclosed. No writing assistance was utilized in the production of this manuscript.

## Supporting information


**Appendix S1** Supporting information
