# Supplementary material for: Impact of older age in patients receiving atezolizumab and bevacizumab for hepatocellular carcinoma
Source: Liver Int. 2022 Sep 2;42(11):2538–47. doi: 10.1111/liv.15405 (PMC9825835; doi:10.1111/liv.15405)
Supplement: Supplementary file 1 — Appendix S1 Supporting information [file LIV-42-2538-s001.docx]

**Supplementary Material**

**Supplementary Table 1.** Effects of older age and prognostic factors on progression-free survival after atezolizumab and bevacizumab in univariate and multivariate Cox regression models

|  | **Univariate models** | | **Multivariable models** | |
| --- | --- | --- | --- | --- |
|  | Hazard Ratio  (95% CI) | *p* value | Hazard Ratio  (95% CI) | *p* value |
| **Age ≥ 65 years** | 0.90 (0.54-1.51) | 0.69 | 1.11 (0.54-1.92) | 0.72 |
| **BCLC Stage (C vs A or B)** | 1.56 (0.92-2.64) | 0.10 | 0.93 (0.49-1.75) | 0.82 |
| **CTP Class (B vs A)** | 2.29 (1.35-3.87) | 0.002 | 1.89 (1.05-3.40) | **0.03** |
| **Tumour size > 7cm** | 1.28 (0.76-2.15) | 0.36 | 1.12 (0.65-1.95) | 0.68 |
| **MVI** | 2.30 (1.38-3.82) | 0.001 | 1.97 (1.01-3.83) | **0.046** |
| **Metastatic Disease** | 1.03 (0.60-1.75) | 0.92 | 1.20 (0.68-2.14) | 0.53 |
| **AFP > 400 ng/dL** | 1.35 (0.81-2.26) | 0.24 | 1.13 (0.66-1.94) | 0.67 |
| **HCV vs other aetiologies** | 1.46 (0.88-2.42) | 0.14 | 1.62 (0.96-2.74) | 0.07 |

**Abbreviations:** 95% CI – 95% Confidence Interval; BCLC – Barcelona Clinic Liver Cancer; CTP – Child-Turcotte-Pugh; MVI – Macrovascular invasion; AFP – alpha-fetoprotein; HCV – Hepatitis C virus;

**Supplementary Table 2.** Baseline characteristics of study population stratified by age 75 years

|  | **All patients**  (n=191) | **Younger age**  (n=142) | **Older age**  (n=49) | ***p value*** |
| --- | --- | --- | --- | --- |
| **Centre** |  |  |  |  |
| Germany | 30 (15.7) | 22 (15.5) | 8 (16.3) | <0.001 |
| Austria | 12 (6.3) | 7 (4.9) | 5 (10.2) |  |
| United Kingdom | 15 (7.9) | 14 (9.9) | (2.0) |  |
| Italy | 12 (6.3) | 10 (7.0) | 2 (4.1) |  |
| United States of America | 60 (31.4) | 56 (39.4) | 4 (8.2) |  |
| Japan | 51 (26.7) | 24 (16.9) | 27 (55.1) |  |
| Taiwan | 11 (5.8) | 9 (6.3) | 2 (4.1) |  |
| **Median Age (IQR)** | 68.4 (61.8-75.2) | 64.8 (59.3-69.7) | 80.1 (78.0-84.0) | <0.001 |
| **Male Sex** | 161 (84.3) | 123 (86.6) | 38 (77.6) | 0.10 |
| **Risk factors for chronic liver disease** |  |  |  |  |
| Non-alcoholic fatty liver disease | 25 (13.1) | 12 (8.5) | 13 (26.5) | 0.001 |
| Alcohol related | 73 (38.2) | 57 (40.1) | 16 (32.7) | 0.35 |
| Hepatitis B infection | 37 (19.4) | 34 (23.9) | 3 (6.1) | 0.01 |
| Hepatitis C infection | 72 (37.7) | 61 (43.0) | 11 (22.5) | 0.01 |
| Other | 12 (8.6) | 8 (6.8) | 4 (18.2) | 0.08 |
| **Child-Turcotte-Pugh class** |  |  |  |  |
| A | 147 (77.0) | 106 (74.7) | 41 (83.7) | 0.20 |
| B | 44 (23.0) | 36 (25.4) | 8 (16.3) |  |
| **Baseline Liver Disease** |  |  |  |  |
| Ascites | 57 (29.8) | 46 (32.4) | 11 (22.5) | 0.19 |
| Hepatic encephalopathy | 11 (5.8) | 11 (7.8) | 0 | 0.04 |
| Varices present | 39 (20.4) | 36 (25.4) | 3 (6.1) | 0.004 |
| **Maximum Tumor Diameter (cm)** | 6.8 (4.9) | 7.11 (5.0) | 6.07 (0.65) | 0.21 |
| **Macrovascular Invasion (MVI)** | 78 (40.8) | 64 (45.1) | 14 (28.6) | 0.04 |
| **Extrahepatic spread (EHS)** | 72 (37.7) | 55 (38.7) | 17 (34.7) | 0.62 |
| **AFP (ng/dL)** |  |  |  |  |
| ≤400 | 126 (66.0) | 95 (66.9) | 31 (63.3) | 0.64 |
| >400 | 65 (34.0) | 47 (33.1) | 18 (36.7) |  |
| **ECOG-PS** |  |  |  |  |
| 0 | 119 (63.0) | 89 (63.6) | 30 (61.2) | 0.79 |
| 1 | 64 (33.9) | 46 (32.9) | 18 (36.7) |  |
| 2 | 6 (3.2) | 5 (3.6) | 1 (2.0) |  |
| **Barcelona Clinic Liver Cancer Stage** |  |  |  |  |
| A | 7 (3.7) | 4 (2.9) | 3 (6.1) | 0.08 |
| B | 68 (36.2) | 45 (32.4) | 23 (46.9) |  |
| C | 113 (60.1) | 90 (64.8) | 23 (46.9) |  |
| **ALBI Score** | -2.2 (0.6) | -2.2 (0.6) | -2.3 (0.6) | 0.63 |
| Grade 1 | 67 (35.1) | 46 (32.4) | 21 (42.9) | 0.19 |
| Grade 2 | 106 (55.5) | 83 (58.5) | 23 (46.9) | 0.16 |
| Grade 3 | 18 (9.4) | 13 (9.2) | 5 (10.2) | 0.83 |
| **Laboratory** |  |  |  |  |
| Serum albumin (g/L) | 35.8 (5.9) | 35.9 (6.0) | 35.5 (5.7) | 0.66 |
| Bilirubin (µmol/L) | 23.1 (40.9) | 25.8 (46.8) | 15.4 (9.6) | 0.13 |
| Platelet count (x10^9^/L) | 181.7 (97.9) | 182.6 (105.2) | 179.1 (73.3) | 0.83 |
| **Previous locoregional treatment** |  |  |  |  |
| Resection | 44 (23.0) | 28 (19.7) | 16 (32.6) | 0.18 |
| Radiofrequency ablation | 38 (19.9) | 26 (18.3) | 12 (24.5) | 0.35 |
| Transarterial chemoembolization | 57 (29.8) | 44 (31.0) | 13 (26.5) | 0.56 |
| Y90 | 21 (11.0) | 19 (13.4) | 2 (4.1) | 0.07 |
| External Beam Radiotherapy | 6 (3.1) | 5 (3.5) | 1 (2.0) | 0.61 |
| **Median immunotherapy duration** (IQR, *months)* | 3.5 (1.5-7.7) | 4.0 (1.6-8.0) | 2.8 (1.4-6.7) | 0.31 |

**Notes**: n (%) for discrete variables; mean ± standard deviation for continuous variables

**Abbreviations:** AFP – alpha-fetoprotein; ECOG-PS – Eastern Cooperative Oncology Group Performance Status.

**Supplementary Table 3.** Effects of older age and prognostic factors on overall survival after atezolizumab and bevacizumab in univariate and multivariate Cox regression models for age 75 years and older

|  | **Univariate models** | | **Multivariable models** | |
| --- | --- | --- | --- | --- |
|  | Hazard Ratio  (95% CI) | *p* value | Hazard Ratio  (95% CI) | *p* value |
| **Age ≥ 75 years** | 0.96 (0.52-1.78) | 0.90 | 1.18 (0.60-2.31) | 0.63 |
| **BCLC Stage (C vs A or B)** | 1.50 (0.89-2.52) | 0.13 | 0.96 (0.52-1.77) | 0.90 |
| **CTP Class (B vs A)** | 3.01 (1.77-5.13) | <0.001 | 2.52 (1.40-4.56) | 0.002 |
| **Tumour size > 7cm** | 1.30 (0.77-2.20) | 0.32 | 1.13 (0.66-1.93) | 0.66 |
| **PVT** | 2.51 (1.15-4.18) | <0.001 | 1.85 (0.99-3.48) | 0.05 |
| **Metastatic Disease** | 0.80 (0.47-1.36) | 0.41 | 0.94 (0.54-1.66) | 0.84 |
| **AFP > 400 ng/dL** | 1.32 (0.79-2.19) | 0.29 | 1.17 (0.68-1.99) | 0.57 |
| **HCV vs other aetiologies** | 1.51 (0.91-2.50) | 0.11 | 1.78 (1.03-3.06) | 0.04 |

**Abbreviations:** 95% CI – 95% Confidence Interval; BCLC – Barcelona Clinic Liver Cancer; CTP – Child-Turcotte-Pugh; PVT – Portal vein tumour; AFP – alpha-fetoprotein; HCV – Hepatitis C virus;

**Supplementary Table 4.** Effects of older age and prognostic factors on progression-free survival after atezolizumab and bevacizumab in univariate and multivariate Cox regression models for age 75 years and older

|  | **Univariate models** | | **Multivariable models** | |
| --- | --- | --- | --- | --- |
|  | Hazard Ratio  (95% CI) | *p* value | Hazard Ratio  (95% CI) | *p* value |
| **Age ≥ 75 years** | 0.85 (0.46-1.57) | 0.59 | 1.02 (0.52-1.99) | 0.96 |
| **BCLC Stage (C vs A or B)** | 1.56 (0.92-2.64) | 0.10 | 0.92 (0.49-1.73) | 0.80 |
| **CTP Class (B vs A)** | 2.29 (1.35-3.87) | 0.002 | 1.89 (1.05-3.40) | 0.03 |
| **Tumour size > 7cm** | 1.28 (0.76-2.15) | 0.36 | 1.10 (0.64-1.89) | 0.73 |
| **PVT** | 2.30 (1.38-3.82) | 0.001 | 1.94 (1.00-3.77) | 0.049 |
| **Metastatic Disease** | 1.03 (0.60-1.75) | 0.92 | 1.20 (0.67-2.14) | 0.54 |
| **AFP > 400 ng/dL** | 1.35 (0.81-2.26) | 0.24 | 1.15 (0.67-1.98) | 0.62 |
| **HCV vs other aetiologies** | 1.46 (0.88-2.42) | 0.14 | 1.60 (0.93-2.76) | 0.09 |

**Abbreviations:** 95% CI – 95% Confidence Interval; BCLC – Barcelona Clinic Liver Cancer; CTP – Child-Turcotte-Pugh; PVT – Portal vein tumour; AFP – alpha-fetoprotein; HCV – Hepatitis C virus;

**Supplementary Table 5.** Best radiological response evaluated per RECIST criteria version 1.1 stratified by age 75 years

|  | **All patients**  (n=163^a^) | **Younger age**  (n=118) | **Older age**  (n=45) | ***p value*** |
| --- | --- | --- | --- | --- |
| Complete Response | 0 | 0 | 0 |  |
| Partial Response | 40 (24.5) | 27 (22.9) | 13 (28.9) | 0.43 |
| Stable Disease | 79 (48.5) | 62 (52.5) | 17 (37.8) | 0.09 |
| Progressive Disease Rate | 44 (27.0) | 29 (24.6) | 15 (33.3) | 0.26 |

**Notes**: ^a^Radiological response was assessed in 163 patients (85.3%);

**Abbreviations:** RECIST, response evaluation criteria in solid tumours.

**Supplementary Table 6.** Atezolizumab and bevacizumab treatment-related adverse events stratified by age 75 years

|  | **All patients**  (n=191) | **Younger age**  (n=142) | **Older age**  (n=49) | ***p value*** |
| --- | --- | --- | --- | --- |
| **Any grade trAEs** | 127 (66.5) | 101 (71.1) | 49 (53.1) | 0.02 |
|  |  |  |  |  |
| **Grade≥3^a^ trAEs** | 39 (20.4) | 27 (19.0) | 12 (24.5) | 0.41 |
| Atezolizumab-related | 15 (7.9) | 11 (7.8) | 4 (8.2) | 0.93 |
| Bevacizumab-related | 26 (13.6) | 17 (12.0) | 9 (18.4) | 0.26 |
|  |  |  |  |  |
| **trAEs requiring drug discontinuation** | 12 (6.3) | 10 (5.2) | 2 (4.1) | 0.40 |
|  |  |  |  |  |
| **Atezolizumab trAEs** |  |  |  |  |
| Overall | 83 (43.5) | 70 (49.3) | 13 (26.5) | 0.001 |
| Fatigue | 31 (16.2) | 26 (18.3) | 5 (10.2) | 0.18 |
| Hepatotoxicity | 28 (14.7) | 22 (15.5) | 6 (12.2) | 0.58 |
| Skin toxicity | 9 (4.7) | 6 (4.2) | 3 (6.1) | 0.59 |
| Colitis | 24 (12.6) | 19 (13.4) | 5 (10.2) | 0.56 |
| Thyroid dysfunction | 9 (4.7) | 8 (5.6) | 1 (2.0) | 0.31 |
| Pneumonitis | 4 (2.1) | 4 (2.8) | 0 | 0.24 |
|  |  |  |  |  |
|  |  |  |  |  |
| **Bevacizumab trAEs** |  |  |  |  |
| Overall | 83 (43.5) | 64 (45.1) | 19 (38.8) | 0.44 |
| Bleeding | 20 (10.5) | 7 (12.0) | 3 (6.1) | 0.25 |
| Hypertension | 44 (23.0) | 33 (23.2) | 11 (22.5) | 0.91 |
| Proteinuria | 38 (19.9) | 29 (20.4) | 9 (18.4) | 0.76 |
| Thrombosis | 10 (5.2) | 7 (4.9) | 3 (6.1) | 0.75 |

**Notes**: ^a^graded as per the National Cancer Institute Common Terminology Criteria for Adverse Events (CTCAE)

**Abbreviations:** trAE – treatment-related adverse event.

**Supplementary fIGURES**


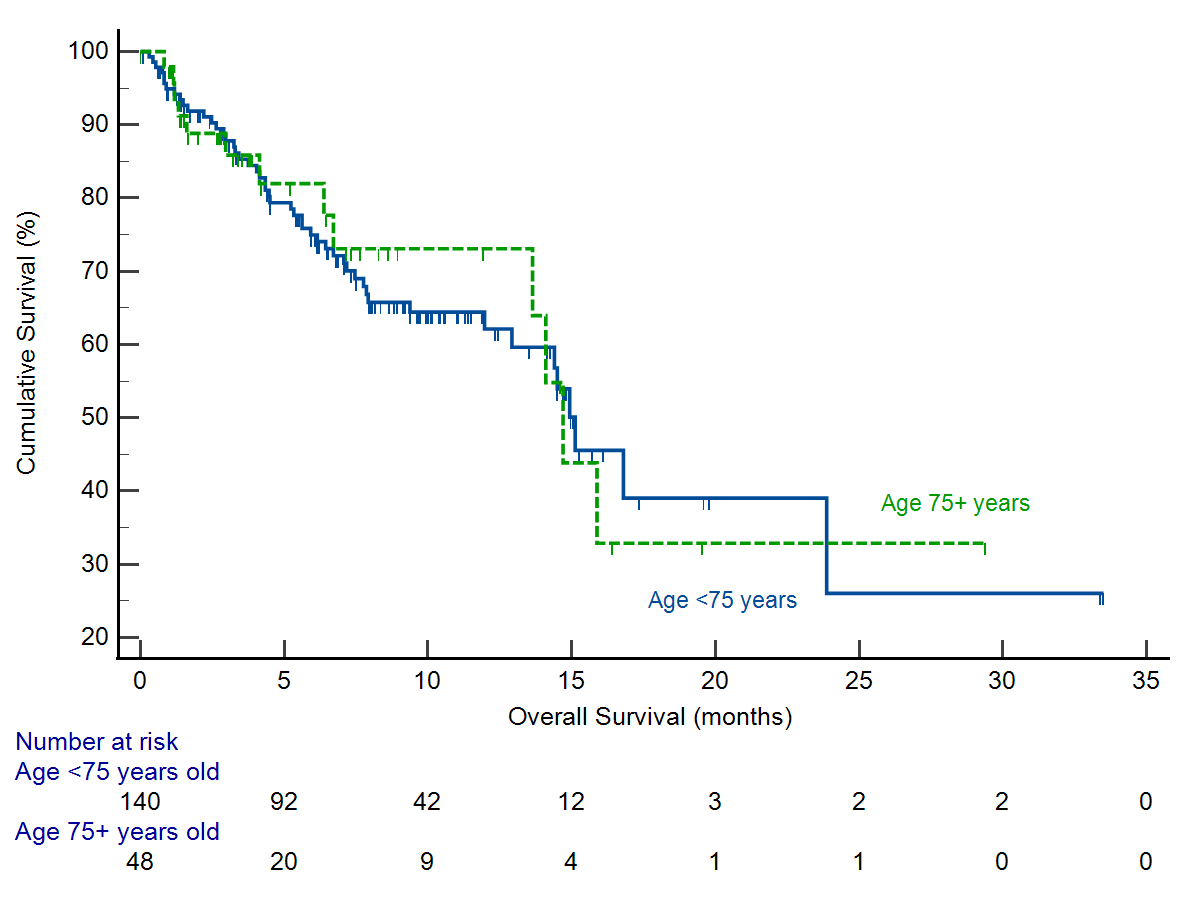
**Supplementary Figure 1.** Kaplan-Meier curve showing overall survival (months) for older age and younger age patients with unresectable hepatocellular carcinoma patients after atezolizumab plus bevacizumab administration stratified by 75 years old


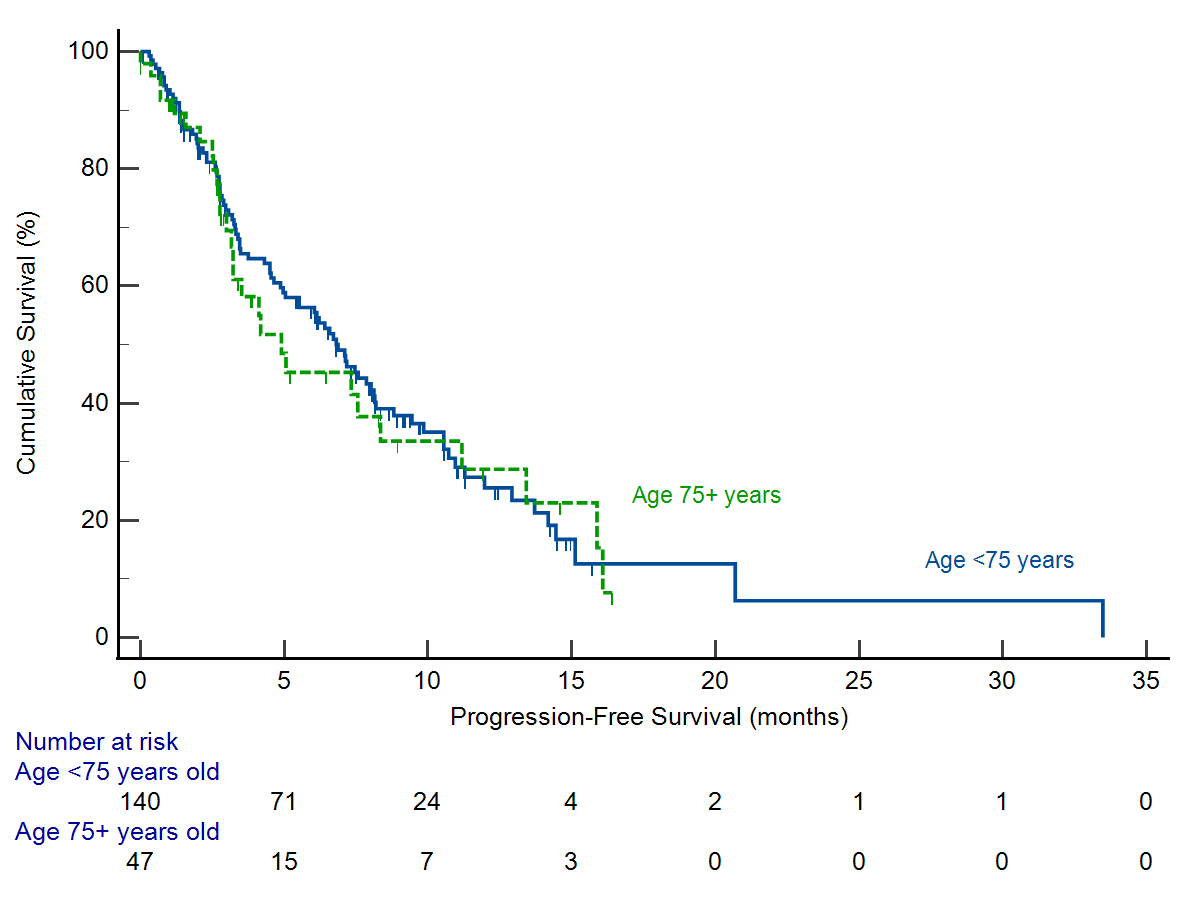
**Supplementary Figure 2.** Kaplan-Meier curve showing progression-free survival (months) for older age and younger age patients with unresectable hepatocellular carcinoma patients after atezolizumab plus bevacizumab administration stratified by 75 years old
